# A rasch analysis of the Manchester foot pain and disability index

**DOI:** 10.1186/1757-1146-2-29

**Published:** 2009-10-30

**Authors:** Sara Muller, Edward Roddy

**Affiliations:** 1 Arthritis Research Campaign National Primary Care Centre, Primary Care Sciences, Keele University, Keele, Staffordshire, ST5 5BG, UK

## Abstract

**Background:**

There is currently no interval-level measure of foot-related disability and this has hampered research in this area. The Manchester Foot Pain and Disability Index (FPDI) could potentially fill this gap.

**Objective:**

To assess the fit of the three subscales (function, pain, appearance) of the FPDI to the Rasch unidimensional measurement model in order to form interval-level scores.

**Methods:**

A two-stage postal survey at a general practice in the UK collected data from 149 adults aged 50 years and over with foot pain. The 17 FPDI items, in three subscales, were assessed for their fit to the Rasch model. Checks were carried out for differential item functioning by age and gender.

**Results:**

The function and pain items fit the Rasch model and interval-level scores can be constructed. There were too few people without extreme scores on the appearance subscale to allow fit to the Rasch model to be tested.

**Conclusion:**

The items from the FPDI function and pain subscales can be used to obtain interval level scores for these factors for use in future research studies in older adults. Further work is needed to establish the interval nature of these subscale scores in more diverse populations and to establish the measurement properties of these interval-level scores.

## Background

It has been estimated that the prevalence of foot pain in community dwelling adults aged 65 years and over is between 20 and 42% [1-4] and foot pain is known to contribute to locomotor disability [1-9]. However, research has been hampered by the lack of an instrument with which to measure foot-related disability. The Manchester Foot Pain and Disability Index (FPDI) [10] could potentially fill this gap. The FPDI is a self-complete questionnaire consisting of 19-items, each of which has three possible response categories: "none of the time", "on some days" or "on most/every day(s)" [10]. These items were developed from interviews with people attending foot clinics for treatment who were asked open-ended questions about pain, disability, activity limitation and footwear [10]. In the development of the questionnaire, it was suggested that the two items relating to work and leisure be removed, as they might not relevant to all populations. Exploratory factor analysis then suggested that the remaining 17 items could be formed into four subscales: functional problems (10 items), two pain intensity constructs (2 items and 3 items) and personal appearance (2 items). The authors suggested that the two pain intensity subscales be combined to give 3 subscales in total (function, pain intensity, appearance) over the 17 items [10].

In the original development of the FPDI, Garrow et al [10] suggested that a simple score could be derived for each subscale. However, in their subsequent population survey, they defined disabling foot pain as present if at least one of the 17 pain intensity, function or appearance items occurred on at least "some days" in the past month [6]. Other authors have also used this approach [11,12]. A further study by Cook et al used exploratory factor analysis to derive two subscales (foot and ankle function (9 items) and pain and appearance (7 items)) for the FPDI having deleted one item ("My feet are worse in the morning") because it did not load on to either of the factors [13]. These authors called this the Modified Manchester FPDI. However, a more recent study by Roddy et al [14] undertook confirmatory factor analysis to verify the original three subscales of Garrow et al in the 17 items (function (10 items), pain (5 items) and appearance (2 items)) [10] and demonstrated the validity and reliability of a new definition of disabling pain that required the occurrence of a problem on at least one of the ten items on the function subscale on "most/every day(s)" in the past month. In this latter study [14], the definition of disabling pain was modified, as using Garrow's definition [6], 98% of older adults with foot pain were classified as having disabling foot pain.

Each of the definitions described above produces a dichotomous evaluation of disabling foot pain, that is, disability is either present or absent. In reality, the disability caused by foot pain will be displayed along a continuum, with different people displaying differing degrees of disability. Garrow et al proposed that, using a simple scoring system, individual scores for each of the three subscales could be generated to produce an overall index of disability [10] and then, in a later study, suggested summating scores for each of the subscales expressed as a percentage ("none of the time" = 0, "on some days" = 1, "on most/every day(s)" = 2) [6]. This scoring system was used subsequently by Menz et al to produce a total FPDI score ranging from 0 to 34 in addition to subscale scores [12]. Other authors have used a different scoring system ("none of the time" = 1, "on some days" = 2, "on most/every day(s)" = 3) to produce a total score ranging from 0 to 51 and individual subscale scores [13,15]. However, these summated totals were not suitable to correctly examine changes in score over time, or differences in scores between groups, because they were not shown to be unidimensional and were not of an interval-level, i.e. where a difference of, say, two points on the score is equivalent at all points along the continuum [16,17].

The only way to derive interval-level scores from ordinal item responses such as those in the FPDI is through the use of the Rasch unidimensional measurement model [18,19]. The objective of this study was to employ the Rasch model to assess the performance of the three FPDI subscales and to attempt to derive interval level subscale scores for each of the three factors of the FPDI [10,14].

## Methods

### Study sample

Data for these analyses were collected in a pilot study for the North Staffordshire Osteoarthritis Project (NorStOP). The methodology for mailing Health Survey and Regional Pains Survey questionnaires in this pilot study replicated that used in the main survey, details of which have been published previously [20]. In summary, the design of the study was a two-stage cross-sectional postal survey of adults aged 50 years and over using self-complete questionnaires. A random sample of 1000 people was selected from a single general practice from the North Staffordshire General Practice Research Network. Stage 1 of the survey consisted of a Health Survey questionnaire. Responders to this questionnaire who reported foot pain in the last 12 months and gave consent to be contacted again were then sent Stage 2, a Regional Pain Survey questionnaire, which gathered more detailed information on their foot problems, including the Manchester Foot Pain and Disability Index [10].

### The Rasch model

The Rasch model has been described in detail elsewhere [21-23]. Briefly, a logistic function is used to relate the difficulty of an item to the ability of a person in order to obtain an interval-level score. Estimates of item difficulty and person ability are independent of each other [24], making the scale score relatively distribution-free [21]. The following sections describe characteristics explored within the Rasch model and how they are evaluated.

#### The model

The partial credit Rasch model [25] was used to create a separate score for each subscale of the FPDI (function, pain, appearance) using the RUMM2020 Rasch analysis package [26].

Threshold plots were inspected to ensure that response categories were ordered as would be expected (i.e. that respondents considered endorsing an item on "some days" to represent more disability than endorsing an item "none of the time", but less disability than endorsing it on "most/every day(s)").

#### Unidimensionality

It is essential that any scale is measuring only a single construct [27]. To ensure that the FPDI scales were unidimensional, a principal components analysis of the residuals was performed. The aim of this is to identify patterns of the residuals once the 'Rasch factor' has been extracted. This is important in order to identify any subsets of items that may be loading together, and therefore may represent a different construct. The absence of any meaningful pattern in the residuals is deemed to support the assumption of local independence of the items. In order to explore this, the two most different groups of items (i.e. those whose fit residuals load negatively and those that load positively onto the first component) were ascertained from the principal components analysis. These two sets of items produce the most different estimates of person location. Using these two sets of person locations, independent sample t-tests were conducted to assess the proportion of people in which there was a significant difference between the person locations based on the two groups of items. In order to accept that all of the items in a scale were measuring the same underlying construct, it was required that no more than 5% of these t-tests result in a p-value < 0.05 [27].

#### Response dependency

Response dependency occurs when the response to one item determines the response to another item [28]. For example, if a person can walk a mile, they must also be able to walk half a mile. Response dependency was assessed via the residual correlations between items, with a positive correlation noticeably higher than other correlations [29] taken to indicate dependency.

#### Item fit

Overall item fit was examined via the mean item fit residual. This value was expected to be approximately zero, with a standard deviation (SD) of one if the data fit the Rasch model.

The fit of individual items was examined in three different ways; the individual item fit residuals, a chi-square test and an F-test, giving three perspectives on the fit of the items [30]. The item fit residual was expected to be in the range -2.5 to +2.5 [31]. For the chi-square and F-tests, the null hypothesis was that the data were a good fit to the Rasch model. Therefore, p-values < 0.05 indicated poor fit of the item to the model. The F-test is generally more sensitive to departures from the Rasch model than the chi-square test [29]. Bonferroni adjustments [32] were made to the significance levels for the chi-square and F-tests, based on the number of items in the scale, to account for multiple testing. Therefore the critical values for each of the scales were: function 0.005, pain 0.01 and appearance 0.025.

#### Person fit

Overall fit of persons to the model was examined via the mean person fit residual. As with the item fit residual, if the data fit the Rasch model, the mean value was expected to be approximately zero with a standard deviation of one.

Individual person fit was assessed via the individual person fit residuals. A residual value less than -2.5 was considered indicative of a purer Guttman response pattern [33] than expected by the probabilistic Rasch model and was not regarded as problematic. A residual value greater than +2.5 was considered to be indicative of an unexpected response pattern under the Rasch model and was further investigated with a view to removing such persons from the sample [30].

#### Overall fit to the Rasch model

The item-trait interaction statistic is a measure of the overall fit of the data to the Rasch model. A statistically significant result on this chi-square test indicated that the hierarchical ordering of the items was not constant along the latent trait [34] and hence an interval level score has not been created.

#### Differential item functioning

Differential item functioning (DIF) occurs when different groups of respondents (e.g. males and females) respond differently to an individual item, despite having the same level of the underlying trait [30]. This is important because DIF can be considered a breach of unidimensionality and so items displaying substantial DIF were considered for removal from the scale [31].

In these analyses, DIF was assessed by means of a 2-way analysis of variance (ANOVA) for gender and age group (50 to 59 years, 60 to 69 years, 70 years and over) separately. A significant main effect for gender (age group) would indicate uniform DIF, i.e. males and females (different age groups) responded systematically differently to the item in question along the latent trait. A significant interaction effect between gender (age group) and the trait would indicate the presence of non-uniform DIF on this item, i.e. males and females (different age groups) responded differently to the item in question and this difference varied along the continuum of the latent trait. As for the analysis of item fit, the critical values for each of the scales were: function 0.005, pain 0.01 and appearance 0.025 after applying the Bonferroni correction [32].

#### Targeting of the scale

The targeting of the items and persons was assessed by comparing the mean person location to the mean item location (constrained to be zero). A negative mean person location indicates that the average item difficultly is above the average disability of the sample. A positive mean person location indicates that the average item difficulty is above the average disability of the sample. A mean person location of zero indicates that the items and the sample are perfectly targeted.

The Person Separation Index (PSI) was considered as a measure of the ability of the scale to differentiate between people. A value of 0.7 was considered suitable for group comparisons [30].

## Results

### Study sample

Of the 1000 Health Survey questionnaires mailed, 745 completed questionnaires were returned (adjusted response rate 77.3%). Two hundred and seventy-five respondents reported that they had experienced foot pain in the previous year. Two hundred and twenty-three of these provided consent for further contact and were mailed a Regional Pains Survey questionnaire. One hundred and ninety-seven completed questionnaires were received. The initial sample for this study then consisted of 149 people (63% female, mean (SD) age 66.1 (9.5) years) who reported foot pain on both the Health Survey and Regional Pains Survey questionnaires and had answered at least some of the FPDI items. Although a Rasch score can be estimated for those people with extreme scores (i.e. responded "none of the time " or "on most/every day(s)" to all items within a subscale), these people cannot be used in the estimation of model parameters. Hence, having removed those with extreme scores, 131 people were available for the derivation of the function subscale score, 133 for the pain subscale and 36 for the appearance subscale. This sample size for the appearance subscale was considered to be too small to allow assessment of the subscale's properties, and so further analyses of the two appearance items were not undertaken.

### Fit of the data to the Rasch model

Thresholds for all items in the function and pain subscales were ordered as expected.

#### Unidimensionality

Independent t-tests showed the function and pain subscales of the FPDI to be unidimensional with less than five percent of people having different locations at the five percent level (function: 4.6% (95% CI 0.8%, 8.3%); pain: 0.8% (-3.0%, 4.5%)).

#### Response dependency

There were no positive residual correlations noticeably larger than the other correlations in any of the subscales. Correlations were in the range -0.28 to +0.09 for the function subscale and -0.36 to -0.10 for the pain subscale. Hence there was no evidence of response dependency in any of the subscale items.

#### Item fit

Item locations and their standard errors are shown in Table 1. These locations allow the ordering of the items in terms of the difficulty of the tasks to which they pertain. The first item in the function scale is Item 6 (avoid walking on hard or rough surfaces) with a location on the foot function scale of -1.339 logits, i.e. the analysis indicates that walking on rough or hard surfaces is the most difficult task on the scale for people with foot pain to perform and, hence, is avoided by those with even the mildest level of disability, as measured by the FPDI. Item 1 is the last item with a location of +2.166 logits, i.e. the analysis indicates that walking outside is the least difficult task on the scale and, hence, is avoided by only those with very poor function.

**Table 1:** Item locations and fit statistics for the 15 items of the FPDI function and pain subscales

	**Location (SE) (logits)**	**Item fit residual**	**Chi-square probability**	**F-test probability**
*Functioning Subscale*				
1. Avoid walking outside	2.166 (0.223)	-0.141	0.5146	0.6898
2. Avoid walking long distances	-0.963 (0.160)	-0.989	0.3109	0.1625
3. Don't walk in a normal way	-0.082 (0.169)	0.984	0.2666	0.4895
4. Walk slowly	-0.867 (0.164)	-1.050	0.7594	0.4466
5. Have to stop and rest feet	0.115 (0.175)	-0.941	0.3065	0.2227
6. Avoid hard or rough surfaces	-1.339 (0.156)	-0.374	0.6770	0.5616
7. Avoid standing for a long time	-1.058 (0.165)	-1.011	0.5779	0.4415
8. Catch the bus or use the car more often	-0.897 (0.153)	0.809	0.2563	0.3129
9. Need help with housework or shopping	1.760 (0.208)	-1.757	0.1866	0.0210
11. Get irritable when feet hurt	1.165 (0.191)	2.302	0.0416	0.0737
*Pain Subscale*				
10. Do everything with more pain or discomfort	-0.868 (0.148)	1.682	0.2000	0.2046
14. Constant pain in feet	-0.184 (0.147)	-1.207	0.0275	0.0030*
15. Feet are worse in the morning	0.513 (0.150)	0.318	0.1979	0.2203
16. Feet more painful in the evening	-0.201 (0.151)	-0.478	0.2735	0.1327
17. Get shooting pains in feet	0.739 (0.154)	1.237	0.5830	0.6318

* Significant misfit after Bonferroni correction

**Figure 1:**
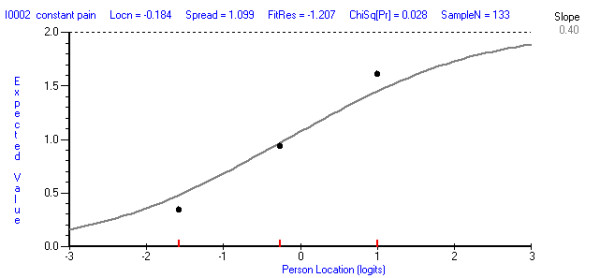
**Item characteristic curve for Item 14 (constant pain in feet)**.

Overall item fit as described by the mean (SD) item fit residual was good for the function and pain subscales (function: -0.217 (1.233); pain: 0.308 (1.187)). Table 1 shows the fit of the individual items. There was no misfit as measured by the item residuals or the chi-square fit statistic in either of the subscales, after applying the Bonferroni correction. In the pain scale, there was misfit on the F-test after Bonferroni correction (p = 0.0030) on the item relating to having constant pain. Figure 1 shows that this item is slightly over discriminating.

#### Person fit

Overall person fit as described by the mean person fit (SD) residual was reasonable in both subscales (function: -0.312 (0.944); pain: -0.216 (0.999)).

In the function scale, three individuals had a person fit residual outside the range -2.5 to +2.5. In the pain scale, one person had a residual outside this range. With one exception, the residuals outside the acceptable range were negative and hence indicative of a purer Guttman pattern than expected by the Rasch model. In the function scale, one person had a residual greater than +2.5 because of a response pattern that was unexpected under the Rasch model. This person was removed from the analysis, but this did not change the overall fit of the data to the Rasch model. Hence it was decided to retain this person in the sample.

#### Overall model fit

The assumption of invariance along the latent trait held in both of the subscales, as evidenced by the item-trait interaction statistics (function: Χ^2 ^= 23.543, df = 20, p = 0.2629; pain: Χ^2 ^= 17.318, df = 10, p = 0.0676).

#### Differential Item Functioning

There was no DIF by gender on either of the subscales after Bonferroni correction (Table 2).

**Table 2:** Differential item functioning by gender and age for the 15 items of the FPDI pain and function subscales

**Item**	**Differential item functioning: gender**		**Differential item functioning: age group**	
	**Uniform^a^**	**Non-uniform^b^**	**Uniform^a^**	**Non-uniform^b^**
*Functioning*				
1. Avoid walking outside	0.2073	0.6787	0.7507	0.0786
2. Avoid walking long distances	0.2544	0.8759	0.7729	0.3730
3. Don't walk in a normal way	0.9714	0.0968	0.0895	0.9878
4. Walk slowly	0.0161	0.3659	0.6014	0.3262
5. Have to stop and rest feet	0.1247	0.5087	0.7701	0.3262
6. Avoid hard or rough surfaces	0.0673	0.4546	0.0014*	0.9992
7. Avoid standing for a long time	0.5544	0.8868	0.6841	0.1632
8. Catch the bus or use the car more often	0.4011	0.0614	0.9277	0.2543
9. Need help with housework or shopping	0.9402	0.9359	0.1205	0.8793
11. Get irritable when feet hurt	0.5657	0.2936	0.4229	0.9999
*Pain*				
10. Do everything with more pain or discomfort	0.1000	0.3845	0.6579	0.3527
14. Constant pain in feet	0.6385	0.0736	0.6426	0.5198
15. Feet are worse in the morning	0.4947	0.9999	0.1955	0.1860
16. Feet more painful in the evening	0.0878	0.7780	0.5688	0.8217
17. Get shooting pains in feet	0.4986	0.7282	0.6482	0.8619

^a ^Uniform DIF is assessed by the p-value associated with the main effect term in a 2-way ANOVA; ^b ^Non-uniform DIF is assessed by the p-value associated with the interaction term in a 2-way ANOVA; * Significant misfit after Bonferroni correction

**Figure 2:**
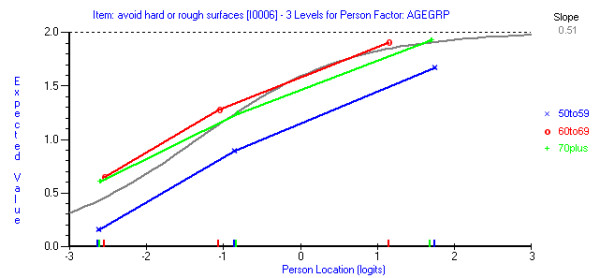
**Differential item functioning for age group in the functioning scale (Item 6, avoid walking on rough or hard surfaces)**.

The age groups used in the DIF analysis were of similar sizes (50 to 59 years, n = 46; 60 to 69 years, n = 47; 70 years and over, n = 56). There was no DIF by age group on the pain subscale as all p-values were greater than 0.01. On the function subscale, there was uniform DIF by age group (p = 0.0014) with those aged 60 years and over more likely to endorse the Item 6 (avoid rough or hard surfaces) than those aged 59 years and under (Figure 2). Attempts were made to correct for this DIF by treating this item separately for those aged 50 to 59 years and those aged 60 years and over. The subscale was also assessed with this item deleted. Neither of these strategies improved overall model fit and so it was decided to retain this item in the functioning subscale in its original form.

#### Targeting

**Figure 3:**
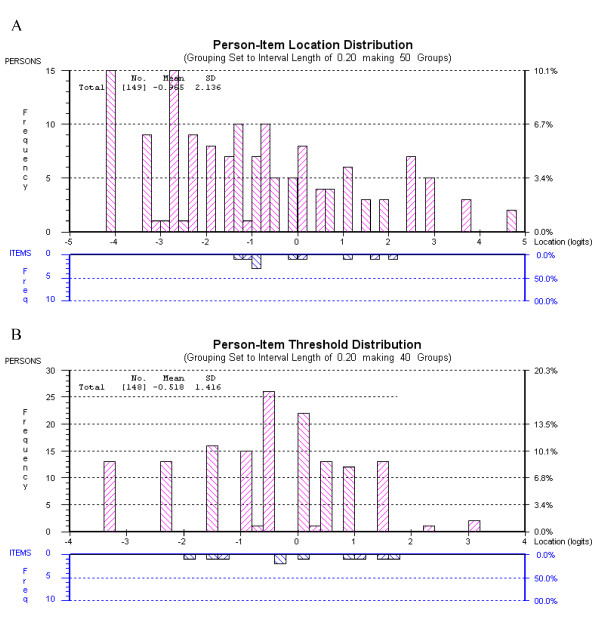
**Person-threshold distribution maps**. A Function subscale. B Pain subscale.

Figure 3 shows that although there are ceiling and floor effects in both the function and pain subscales, the item thresholds are generally spread along the continuum of the traits displayed by the sample. The mean (SD) person locations for the subscales were function: -0.965 (2.136) and pain: -0.522 (1.415). Both subscales have a negative person location, indicating that, the average item difficulty is higher than the average person disability. The pain subscale is better targeted than the function subscale.

The Person Separation Index was acceptable for both subscales (function: 0.915; pain: 0.718), showing a good ability to distinguish between people along the latent traits [30].

## Discussion

The FPDI is a measure of disability arising as a result of foot-pain that has been used in recent epidemiological studies and clinical trials [6,12-15]. In epidemiological studies, the FPDI has been used to produce a dichotomised measure of disability, that is, disability is either present or absent. Recent clinimetric studies and a clinical trial summated the seventeen ordinal items to produce a foot disability score ranging from 0 to 34 [12] or 17 to 51 [13,15]. In the current study, we used the Rasch unidimensional measurement model [19] to obtain interval-level scores for the FPDI pain and function sub-scales.

These analyses have shown that the function and pain subscales of the FPDI are unidimensional and that interval level scores can be obtained from the items of these subscales. It was not possible to assess the measurement properties of the appearance subscale due to the small number of people without extreme responses on this subscale. This is perhaps not surprising, as the appearance subscale consists of only two items, making scoring problematic.

There was some evidence of differential item functioning (DIF) by age on the item relating to avoiding rough and hard surfaces on the function subscale, which could indicate a lack of unidimensionality in this subscale [31]. Attempts were made to correct for this by estimating the item location separately for the younger and older age groups [30]. However, this did not improve the model overall and made the scoring of the subscale more complicated, so this was not carried forward. The item could have been deleted, but this would have changed the subscale from its original form, which was not thought to be desirable. Instead, the item was retained. Furthermore, the original t-test of unidimensionality [27] and the residual correlations between items did not suggest that the function subscale breached unidimensionality. It could be that this item displays DIF because younger people, who are generally still employed, cannot avoid such surfaces or this DIF could have arisen as a result of the small sample size. However, the presence of this DIF and potential reasons for it should be confirmed in an independent sample. It seems likely though that this is a Type I statistical error.

There was also evidence of misfit, from the F-test, for the item relating to having constant pain in the feet but it was not considered necessary to attempt to correct this misfit because of the good fit on the residual and chi-square statistics. It is also known that the F-statistic is very sensitive to departures from fit to the Rasch model [29].

Although this study has investigated the Rasch measurement properties of the FPDI items for the first time, there are several limitations that deserve consideration. The moderate sample size used in this study may have reduced the ability of the analyses to detect misfit to the Rasch model. However, all categories of all items in the pain scale were endorsed by at least 10 people, as were 8 of the items in the function scale (Item 1: 5 people endorse most/every day(s), Item 11: 9 people endorsed most/every day(s)), generally meeting the minimum sample size requirement suggested by Linacre [35]. Although the sample size was only moderate, it had enough statistical power to detect the DIF displayed by Item 6 in the function subscale with respect to age group. Also, in this subscale, the p-value for the overall fit to the Rasch model, described by the item-person interaction chi-square statistic far exceeded the value of 0.05 required in order to find no evidence against the overall fit to the Rasch model.

A further caveat is that this analysis was undertaken in a population of adults aged 50 years and over from a relatively limited geographical area of the UK, and the sample was almost entirely from a white British background. Although Rasch analysis allows a score to be calibrated independently of the distribution of item responses in the sample [21], further analyses should be carried out in younger or more ethnically diverse populations before applying the scoring mechanism more widely. It may also be possible to use the Rasch-scored FPDI in a patient population, where disability would be expected to be more severe, as the population sample in this study had a much lower level of disability than the FPDI subscales were able to measure. Again, further analyses are needed before the FPDI subscales are used in this context and the Foot Impact Scale [36] has already been developing using Rasch analysis for use in populations with rheumatoid arthritis.

In order to be fully useful in clinical practice and research, the score needs to be transferable between populations. There are two main ways in which this could be carried out: the repeated use of the Rasch model or a conversion table. If the Rasch model were to be used in every dataset, a slightly different score range would result on each occasion, but this would allow people to gain a score even if they did not complete all of the items. This option also requires that the clinician or researcher have access to Rasch analysis software. The alternative option is to use a conversion table between a simple sum score of a person's responses (0, 1, 2 for each item) and the Rasch score. This type of table would be simpler, but would mean that those people who do not complete all of the items in the subscale cannot get a score. There is currently little guidance on in the literature on how to transfer a Rasch score between populations, and the final decision on how to do this should be made by the context of each individual study.

The availability of these interval-level subscale scores for function and pain in those with foot pain will allow the severity of disability to be more finely defined than has previously been possible with the dichotomisation of these subscales [6,12,14]. Whilst not necessarily replacing the dichotomous scoring methods suggested by Garrow et al [10] and Roddy et al [14], this interval-level scoring will allow more detailed research, for example looking at progression of disability, than is allowed for by the simple dichotomous measure. Interval-level scores will also allow the use of the FPDI in studies where the aim is to assess change in foot pain and disability severity over time or differences between groups. The interval-level nature of the Rasch person location estimates allows for the sensible investigation of change scores over time and between groups [16,17].

However, with a continuum of disability, it is useful to have a definition of when a score is high enough to classify the individual person as being 'disabled', or when a change in the score over time is clinically significant. Hence, further work is needed to define clinically important changes on these subscales, such that they can be used more meaningfully in longitudinal research into foot disability.

## Conclusion

The FPDI has been confirmed to have two unidimensional subscales in a general population of older adults in the UK: function and pain. These subscales appear to fit the Rasch measurement model and so an interval-level score can be produced for each subscale. Further work is needed to determine this fit in more general populations and to obtain a minimal clinically important change score for the subscales in order to make them more useful in practice. It may also be useful to further examine the two-item appearance subscale of the FPDI, although this may not be worthwhile due to the small number of items in this subscale.

## Competing interests

The authors declare that they have no competing interests.

## Authors' contributions

SM conceived and conducted the analysis and helped in the drafting of the manuscript. ER helped in the drafting of the manuscript. All authors approved the final manuscript.
